# Amputation Predisposes to Higher Cancer-Specific Mortality Than Limb Salvage Surgery in Pediatric Patients With Osteosarcoma of the Limbs: A Propensity Matching Analysis

**DOI:** 10.3389/fsurg.2022.817051

**Published:** 2022-02-09

**Authors:** Jinkui Wang, Jie Tang, Xiaojun Tan, Chenghao Zhanghuang, Liming Jin, Mujie Li, Zhaoxia Zhang, Tao Mi, Dawei He

**Affiliations:** ^1^Department of Urology, Chongqing Key Laboratory of Children Urogenital Development and Tissue Engineering, Chongqing Key Laboratory of Pediatrics, Ministry of Education Key Laboratory of Child Development and Disorders, National Clinical Research Center for Child Health and Disorders, China International Science and Technology Cooperation Base of Child Development and Critical Disorders, Children's Hospital of Chongqing Medical University, Chongqing, China; ^2^Department of Epidemiology, Public Health School, Shenyang Medical College, Shenyang, China

**Keywords:** Osteosarcoma, limbs, amputation, limb salvage, mortality

## Abstract

**Objective:**

With the development of osteosarcoma treatment, limb salvage surgery is gradually replacing amputation as the primary surgical option. Most pediatric osteosarcomas of the limbs undergo limb-salvage surgery. We aimed to use propensity score matching (PSM) analysis test the difference in cancer-specific mortality (CSM) between amputation and limb-salvage surgery in pediatric patients with Osteosarcoma of the limbs. PSM is a statistical method used to deal with data from an Observational Study. The PSM method is designed to reduce the influence of biases and confounding variables to make a more reasonable comparison between experimental and control groups.

**Methods:**

Patient information was downloaded from the SEER (surveillance, epidemiology, and End Results) database from 2004 to 2018. We included all primary pediatric osteosarcoma patients who underwent limb salvage or amputation. Multivariate logistic regression models were used to explore the factors influencing patient choice of amputation. Differences in CSM and other causes of mortality (OSM) between limb salvage and amputation were analyzed using cumulative incidence plots and competitive risk regression tests after 1:1 proportional propensity score matching.

**Results:**

A total of 1,058 pediatric patients with limbs Osteosarcoma were included. Patients who underwent amputations were more likely to be male (OR 1.4, *P* = 0.024) and more likely to have distant metastasis (OR 2.1, *P* < 0.001). Before propensity matching, CSM was 1.4 times higher in patients undergoing amputation than in patients undergoing limb salvage (*P* = 0.017) and 3.4 times higher in OSM (*P* = 0.007). After adjustment for propensity matching, CSM was 1.5 times higher in patients undergoing amputation than in patients undergoing limb salvage (*P* = 0.028), but there was no significant difference in OSM (HR 3.2, *P* = 0.078).

**Conclusions:**

Our results suggested that amputation is associated with a 1.5-fold increase in CSM in pediatric patients with limbs Osteosarcoma. Therefore, in the surgical selection of pediatric patients with Osteosarcoma, limb salvage surgery should be the first choice in the absence of other contraindications.

## Introduction

Osteosarcoma is the most common bone malignancy in children and young adults (1). Previous studies have shown that the incidence varies with age, with an annual incidence of 1.7 per 100,000 children under ten years of age and 8.2 per 100,000 between 10 and 19 years of age (2). Another study found Osteosarcoma in 2–3 per million in all populations and 8–11 per million in people aged 15–19 years (3). Osteosarcomas peak mainly during adolescence between the ages of 10 and 14, with another peak after 80 (4). Osteosarcomas often occur in the metaphysis of long bones, mainly in the lower limbs. Progression of Osteosarcoma is associated with rapid bone growth during adolescence (5). The etiology of Osteosarcoma includes a variety of factors; currently recognized risk factors include ionizing radiation, alkylation agents, chromosomal abnormalities, hereditary retinoblastoma and Paget's disease (6). Current treatment strategies include neoadjuvant chemotherapy, surgical resection of primary tumors and metastases, and additional adjuvant chemotherapy after surgery (7). The treatment of Osteosarcoma has improved over the past 20 years, including neoadjuvant chemotherapy and radiotherapy, but the overall prognosis of Osteosarcoma remains poor (8–10). Osteosarcoma is one of the health hazards in children, accounting for 8.9% of childhood cancer-related deaths. The 5-year survival rate for Osteosarcoma without distant metastasis is 65 to 70% (11), but only 19 to 30% for Osteosarcoma with distant metastasis (12).

Surgery has been recognized as treating primary and recurrent or metastatic Osteosarcoma (13). In the past 30 years, with the development of neoadjuvant chemotherapy, limb salvage therapy has become one of the standard treatment methods for limb osteosarcoma, 90% of patients can be treated with limb salvage therapy, and the success rate of limb salvage is 60~80% (14, 15). Limb salvage surgery can improve patients' quality of life and health psychology, and more than 80% of patients are willing to accept limb salvage surgery (16). However, sometimes amputation is necessary, such as Osteosarcoma of the distal tibia, which is challenging to achieve a negative margin (17). At present, the survival outcome of amputation and limb-salvage surgery is still controversial. Some studies have shown no significant difference in survival rates between amputation and amputation (15, 18), while others have shown significantly higher survival rates for amputation (18, 19). However, the sample size of these articles is small, and there are many selection biases. Although there have been meta-analyses comparing survival rates for amputations and limb salvage, the heterogeneity of the articles makes the results less convincing (20, 21). Therefore, patients from the National Cancer Institute's Surveillance, Epidemiology, and End Results (SEER) program were used for analysis. We performed a propensity score analysis to reduce selection bias and the effect of confounding factors. We created a matched cohort to analyze the difference in survival rates between amputation and salvage surgery for pediatric limb osteosarcoma. Most of the previous studies were single-center studies with fewer patients. In addition, meta-analysis is a synthesis of the results of multiple studies, which has great heterogeneity. Therefore, we used big data analysis to compare survival differences between amputation and limb salvage in children with limb osteosarcoma.

## Methods

### Data Source and Study Population

The patient information was downloaded from the SEER Database, the leading cancer database in the United States, collecting data from 18 cancer registries and covering approximately 28% of the US population (22). The SEER database is public, and patient information is anonymous, so our study does not require ethical approval and informed consent from patients. Our methodology follows the guidelines of the SEER database.

**Figure 1:**
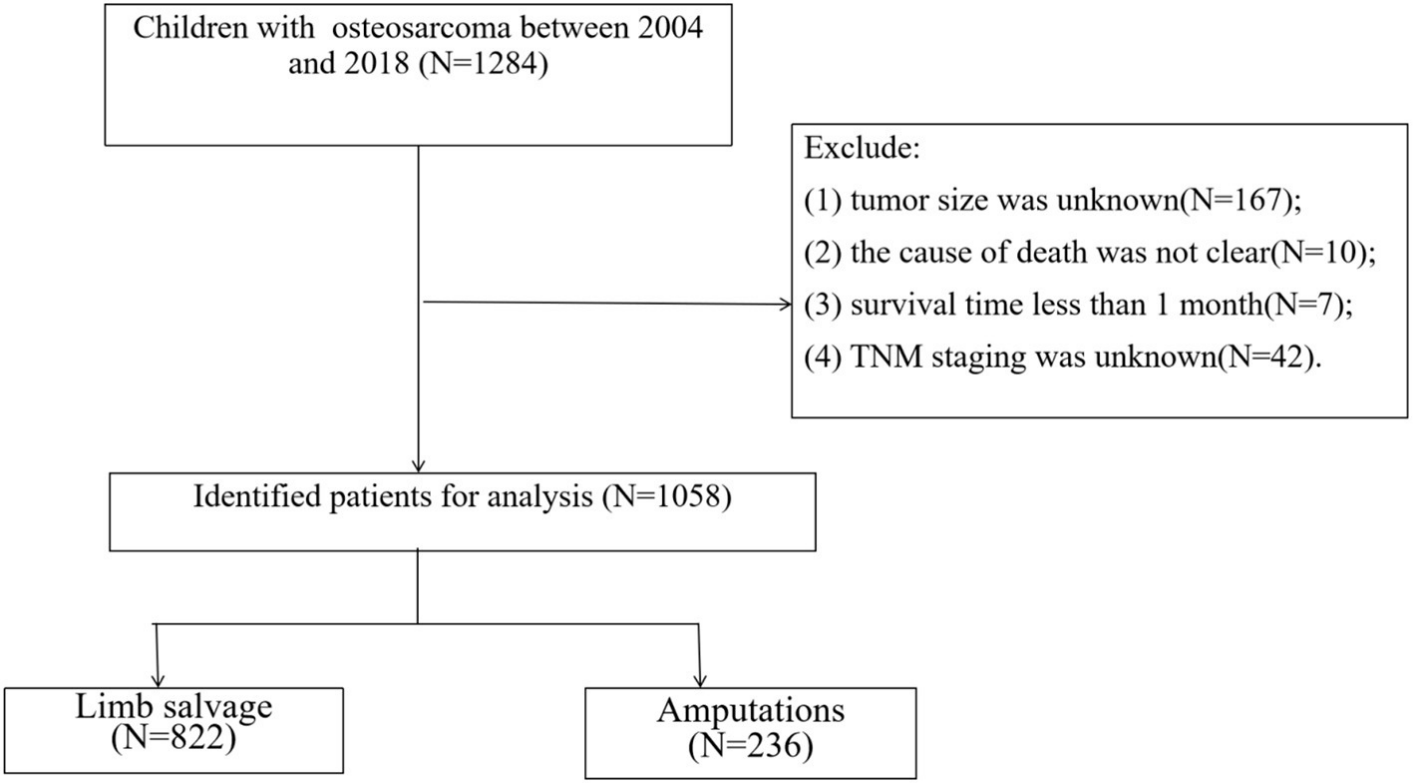
CONSORT (Consolidated Standards of Reporting Trials) diagram of study inclusion criteria.

We collected clinicopathological information from all pediatric osteosarcoma patients from 2004 to 2018. Inclusion criteria: (1) Pathological diagnosis of Osteosarcoma (ICD-O-3 histological codes: 9180-9187 and 9192-9194); (2) The primary site is limb (ICD-O-3 anatomical code: 400-409); (3) The patient was younger than or equal to 18 years old. Exclusion criteria were: (1) tumor size was unknown; (2) the cause of death was not clear; (3) survival time <1 month; (4) TNM staging was unknown. The screening flow chart of all patients is shown in Figure 1.

### Study Variables

In this study, the clinicopathological information of patients was collected. Demographic characteristics include age, sex, race and year of diagnosis; basic tumor information including the pathological grade of tumor, TNM stage of tumor and tumor size; treatment information including surgery, radiotherapy and chemotherapy. To see if the year of diagnosis affected mortality, we divided the patient's years of diagnosis into two categories: 2004-2011 and 2012-2018. Patients were grouped into three racial groups: white, black, and other races (American Indian /AK Indian, Asian/Pacific Islander). There were four tumor grades: grade I (highly differentiated), grade II (moderately differentiated), grade III (poorly differentiated) and grade IV (undifferentiated). Surgical methods include limb salvage and amputation. According to the SEER definition of death, patients die from cancer (CSM) or other causes (OSM). CSM is the death of a patient from cancer, including cancer recurrence and metastasis. OCM is the death of a patient from causes other than cancer, mainly cardiovascular disease, lung disease, etc.

### Statistical Analyses

Categorical variables were described using frequency and proportion, and Continuous variables are described using mean and standard deviation. The proportion difference of categorical variables was analyzed by chi-square test, and the contrast of median was analyzed by *T*-test and Kruskal-Wallis test.

In a cohort of all patients, we used a multivariable logistic regression model to analyze the predictors of choice for amputation and limb-salvage surgery. Variables included age (continuous), gender (male vs. female), race (White vs. black vs. other), year of diagnosis (2004-2011 vs. 2012-2018), laterality (left vs. right), T staging (T1 vs. T2 vs. T3), N staging (N0 vs. N1), M staging (M0 vs. M1), tumor size (continuous).

We used nearest neighbor 1:1 matching in the primary study cohort, which makes the comparison cohort more reliable. The propensity-matched cohort was balanced by age, sex, race, year of diagnosis, tumor grade, laterality, tumor size, TNM stage, chemotherapy, and radiation. Finally, we examined the effects of limb salvage surgery and amputation on CSM and OCM using cumulative incidence plots and multivariable competitive risk regression (CRR) models (23). Competitive risk is that more than one type of event may occur in some cases. The occurrence of one type of event may hinder the observation or change the probability of other kinds of events being observed. The incident prevented another incident under investigation or fundamentally changed the likelihood of that other incident (23). In other words, we used a multivariate CRR model to explore independent risk factors for CSM and OCM in patients. We also compared patient's CSM and OCM differences between amputations and limb salvage under the CRR model.

Then, we performed three sensitivity analyses to optimize the matching results and ensure that our conclusions were reliable. (1) We compared the risk of CSM between amputation and limb salvage using a CRR model in the original cohort. (2) We used a 1:2 and 1:4 propensity match to compare CSM with amputation and limb salvage. (3) We compared the CSM of limb amputation and limb salvage in patients with complete tumor grade information. (4) After inverse probability of treatment weighting, we compared CSM and OCM in patients with amputation and limb salvage.

All statistical analyses were performed using R version 3.4.1 (http://www.r-project.org/) and SPSS software (version 23.0, SPSS, Chicago, IL, USA). We used “Matching,” “nonrandom,” “reshape2,” and “survey” R software packages to create matched sample. We used “foreign,” “survival,” “cmprsk.” and “rms” R software packages to construct a competitive risk model. *P*-values <0.05 were considered statistically significant.

## Results

### Clinical Features

A total of 1,058 patients were enrolled, with 822 patients undergoing limb salvage and 236 patients undergoing amputation. The basic information and clinicopathological features of patients are shown in Table 1. Compared with patients undergoing limb salvage surgery, more patients with amputation were male (61.9% vs. 54.1, *P* = 0.042), with higher T stage (T2-T3, 72.5% vs. 63.7, *P* = 0.034), with distant metastasis (28.8% vs. 15.5, *P* < 0.001), larger tumors (median size 103 vs. 90 mm).

**Table 1:** Sociodemographic and clinical characteristics of patients receiving limb salvage vs. amputation in pediatric osteosarcoma patients.

	**Unmatched**					**Matched**				
	**Overall *N* = 1,058**	**Limb salvage *N* = 822**	**Amputation *N* = 236**	***p*-value**	**SMD**	**Overall *N* = 472**	**Limb salvage *N* = 236**	**Amputation *N* = 236**	***p*-value**	**SMD**
Age [mean (SD)]	12.751 (3.377)	12.809 (3.264)	12.551 (3.745)	0.3009	0.073	12.449 (3.529)	12.347 (3.304)	12.551 (3.745)	0.5319	0.058
Sex				0.042	0.157				0.638	0.052
Male	591 (55.9%)	445 (54.1%)	146 (61.9%)			286 (60.6%)	140 (59.3%)	146 (61.9%)		
Female	467 (44.1%)	377 (45.9%)	90 (38.1%)			186 (39.4%)	96 (40.7%)	90 (38.1%)		
Year of diagnosis				0.289	0.084				0.051	0.189
2004-2011	517 (48.9%)	394 (47.9%)	123 (52.1%)			268 (56.8%)	145 (61.4%)	123 (52.1%)		
2012-2018	541 (51.1%)	428 (52.1%)	113 (47.9%)			204 (43.2%)	91 (38.6%)	113 (47.9%)		
Race				0.054	0.181				0.205	0.165
white	788 (74.5%)	610 (74.2%)	178 (75.4%)			347 (73.5%)	169 (71.6%)	178 (75.4%)		
black	154 (14.6%)	129 (15.7%)	25 (10.6%)			63 (13.3%)	38 (16.1%)	25 (10.6%)		
other	116 (11.0%)	83 (10.1%)	33 (14.0%)			62 (13.1%)	29 (12.3%)	33 (14.0%)		
Grade				0.847	0.092				0.871	0.104
I	17 (1.61%)	14 (1.70%)	3 (1.27%)			7 (1.48%)	4 (1.69%)	3 (1.27%)		
II	28 (2.65%)	22 (2.68%)	6 (2.54%)			13 (2.75%)	7 (2.97%)	6 (2.54%)		
III	247 (23.3%)	198 (24.1%)	49 (20.8%)			106 (22.5%)	57 (24.2%)	49 (20.8%)		
IV	497 (47.0%)	382 (46.5%)	115 (48.7%)			220 (46.6%)	105 (44.5%)	115 (48.7%)		
Unknown	269 (25.4%)	206 (25.1%)	63 (26.7%)			126 (26.7%)	63 (26.7%)	63 (26.7%)		
Laterality				0.996	0.006				1.000	0.008
Left	558 (52.7%)	433 (52.7%)	125 (53.0%)			249 (52.8%)	124 (52.5%)	125 (53.0%)		
Right	500 (47.3%)	389 (47.3%)	111 (47.0%)			223 (47.2%)	112 (47.5%)	111 (47.0%)		
T				0.034	0.195				0.511	0.107
T1	363 (34.3%)	298 (36.3%)	65 (27.5%)			140 (29.7%)	75 (31.8%)	65 (27.5%)		
T2	658 (62.2%)	498 (60.6%)	160 (67.8%)			313 (66.3%)	153 (64.8%)	160 (67.8%)		
T3	37 (3.50%)	26 (3.16%)	11 (4.66%)			19 (4.03%)	8 (3.39%)	11 (4.66%)		
N				0.438	0.049				1.000	0.028
N0	1037 (98.0%)	807 (98.2%)	230 (97.5%)			461 (97.7%)	231 (97.9%)	230 (97.5%)		
N1	21 (1.98%)	15 (1.82%)	6 (2.54%)			11 (2.33%)	5 (2.12%)	6 (2.54%)		
M				<0.001	0.326				0.681	0.047
M0	863 (81.6%)	695 (84.5%)	168 (71.2%)			341 (72.2%)	173 (73.3%)	168 (71.2%)		
M1	195 (18.4%)	127 (15.5%)	68 (28.8%)			131 (27.8%)	63 (26.7%)	68 (28.8%)		
Radiation				0.121	-				0.5738	-
No/Unknown	1036 (97.9%)	808 (98.3%)	228 (96.6%)			459 (97.25)	231 (97.88)	228 (96.61)		
Yes	22 (2.08%)	14 (1.70%)	8 (3.39%)			13 (2.75)	5 (2.12)	8 (3.39)		
Chemotherapy				0.238	-				0.0925	-
No/Unknown	38 (3.59%)	33 (4.01%)	5 (2.12%)			18 (3.81)	13 (5.51)	5 (2.12)		
Yes	1020 (96.4%)	789 (96.0%)	231 (97.9%)			454 (96.19)	223 (94.49)	231 (97.88)		
Tumor.size [mean (SD)]	107.319 (60.855)	105.439 (62.501)	113.869 (54.354)	0.0607	0.144	111.750 (61.667)	109.631 (68.252)	113.869 (54.354)	0.456	0.069

### Original Study Cohort Multivariate Logistic Regression Analysis

In the original cohort of propensity score matching, multivariate Logistic regression analysis was performed on all patients to identify the factors contributing to selecting reasons for limb salvage or amputation. We found that the predictors of patient choice for amputation were male (OR 1.4, *P* = 0.024), other races (OR 1.4, *P* = 0.047), and distant metastasis (OR 2.1, *P* < 0.001). The results of multivariate logistic regression are shown in Table 2.

**Table 2:** Multivariate Logistic regression model to predict limb salvage surgery or amputation in pediatric osteosarcoma patients.

	**OR**	**(5–95% CI)**		***p*-value**
Age	0.965	0.923	1.009	0.12
Sex
Male	Reference			
Female	0.703	0.517	0.955	0.024
Year of diagnosis
2004–2011	Reference			
2012–2018	0.765	0.566	1.032	0.08
Race				0.047
white	Reference			
black	0.64	0.4	1.023	0.062
other	1.365	0.871	2.139	0.175
Laterality
Left	Reference			
Right	0.963	0.715	1.297	0.804
T				0.351
T1	Reference			
T2	1.322	0.892	1.959	0.165
T3	1.46	0.645	3.304	0.364
N
N0	Reference			
N1	1.083	0.394	2.977	0.877
M
M0	Reference			
M1	2.111	1.474	3.023	<0.001
Tumor size	1	0.997	1.003	0.826

### Multivariate Competitive Risk Regression Model After Propensity Score Matching

**Figure 2:**
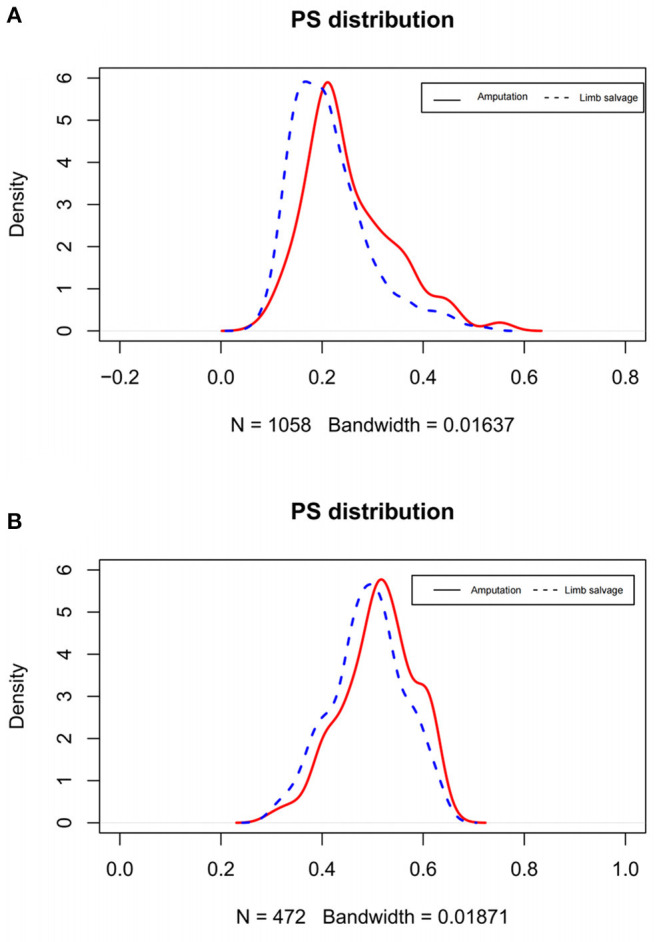
Density plots of propensity score before **(A)** and after matching **(B)**.

**Figure 3:**
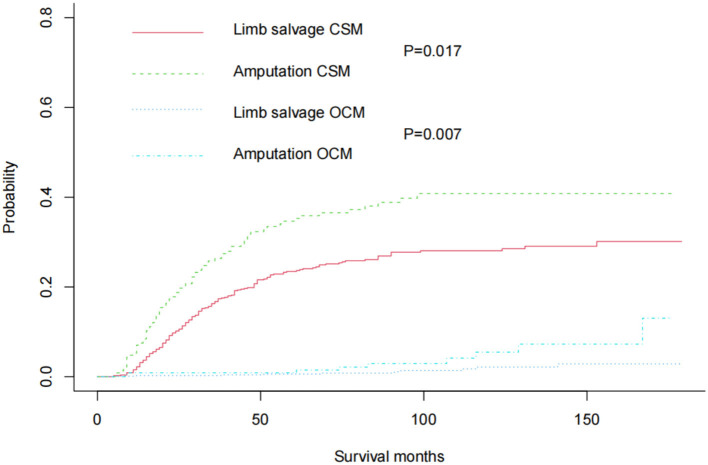
Cumulative incidence plots in the original cohort showed CSM and OCM rates in pediatric patients with Osteosarcoma of the limbs.

**Figure 4:**
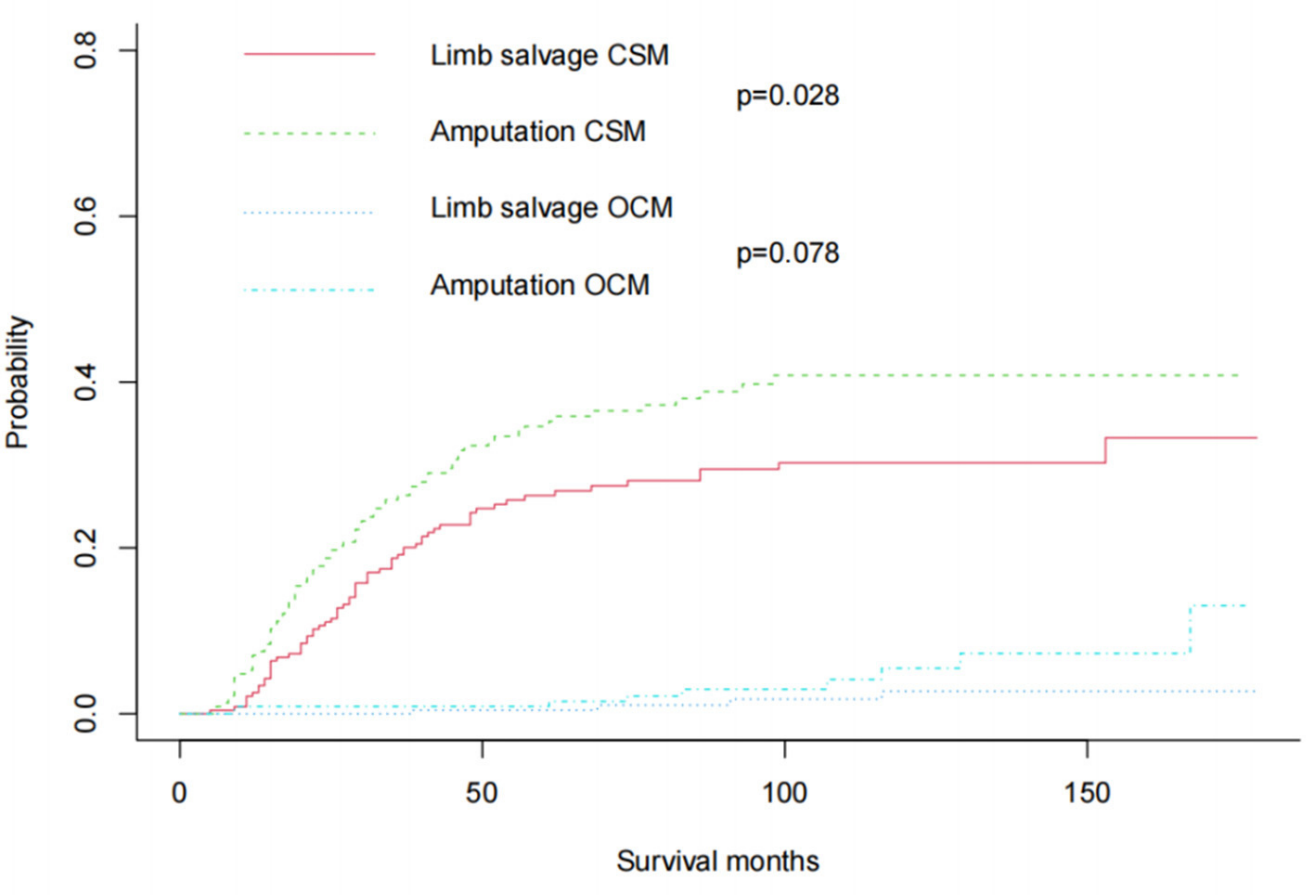
After 1:1 propensity score matching, Cumulative incidence plots showed CSM and OCM rates in pediatric patients with Osteosarcoma of the limbs.

In the original cohort before matching, the CSM of amputation was significantly higher than that of limb salvage (*P* = 0.017). At the same time, the OSM of amputation was also higher than that of limb salvage (*P* = 0.007) (Figure 2). After 1:1 propensity matching, 236 children underwent amputation, and 236 children underwent limb salvage surgery, allowing for subsequent statistical analysis. The propensity score density before and after matching was shown in Figure 3. The patient's information in the two groups was shown in Table 1, and there was no statistical difference in clinicopathological details between the two groups. In the 1:1 propensity matching cohort, the cumulative incidence plot showed a significant difference in CSM between limb salvage surgery and amputation (*P* = 0.028), but no significant difference in OSM (*P* = 0.078) (Figure 4). After propensity matching, multivariable CRR model analysis showed higher CSM in amputation than limb salvage (HR 1.49, *P* = 0.028), but no significant difference in OCM (HR 3.17, *P* = 0.078). Finally, CSM and OSM were analyzed using the multivariate Cox regression model, demonstrating the risk of amputation (Table 3).

**Table 3:** Propensity score-adjusted multivariate Cox regression models predict cancer-specific mortality and other causes mortality in pediatric osteosarcoma patients.

	**Cancer-specific mortality**			**Other cause mortality**		
	**HR**	**(5–95% CI)**	***p*-value**	**HR**	**(5–95% CI)**	***p*-value**
Age	1.028	0.979–1.08	0.272	1.004	0.864–1.167	0.957
Sex				1.132	0.366–3.503	0.83
Male	Reference			Reference		
Female	0.932	0.661–1.312	0.685	1.132	0.366–3.503	0.83
Year of diagnosis
2004–2011	Reference			Reference		
2012–2018	1.071	0.746–1.537	0.709	0.411	0.043–3.917	0.44
Race
white	Reference			Reference		
black	0.843	0.506–1.403	0.511	0.899	0.184–4.384	0.895
other	1.3	0.807–2.092	0.28	0.645	0.079–5.297	0.683
Grade
I	Reference			Reference		
II	0.843	0.506–1.403	0.511	0.882	0-1.82E+143	0.999
III	1.3	0.807–2.092	0.28	2071.991	0-7.63E+120	0.956
IV	0.843	0.506–1.403	0.511	3033.215	0-1.11E+121	0.954
Unknown	1.3	0.807–2.092	0.28	3850.809	0-1.42E+121	0.952
Laterality
Left	Reference			Reference		
Right	0.804	0.571–1.132	0.212	0.683	0.221-2.112	0.508
T
T1	Reference			Reference		
T2	0.942	0.589–1.506	0.802	0.446	0.103-1.921	0.278
T3	1.291	0.581–2.868	0.53	0	0-3.58E+78	0.924
N
N0	Reference			Reference		
N1	1.422	0.601–3.364	0.423	0	0-3.53E+114	0.954
M
M0	Reference			Reference		
M1	3.347	2.34–4.786	<0.001	2.689	0.602–12.021	0.195
Surgery
Limb salvage	Reference			Reference		
Amputation	1.525	1.097–2.121	0.012	3.171	0.972–10.342	0.056
Tumor size	1.003	1–1.005	0.037	1.001	0.99–1.012	0.839

### Sensitivity Analyses

**Figure 5:**
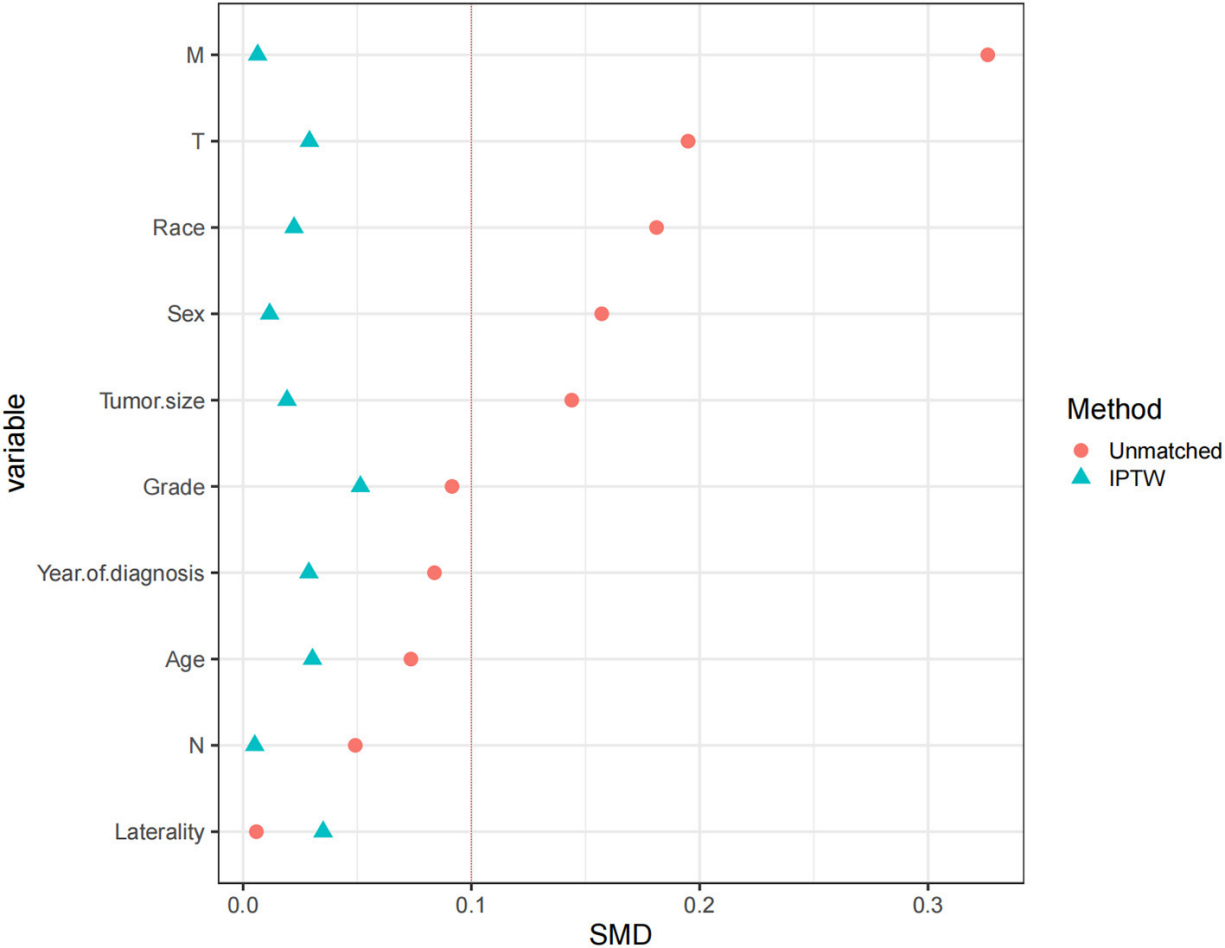
Standardized mean difference distribution of patients after the inverse probability of treatment weighting matching.

**Figure 6:**
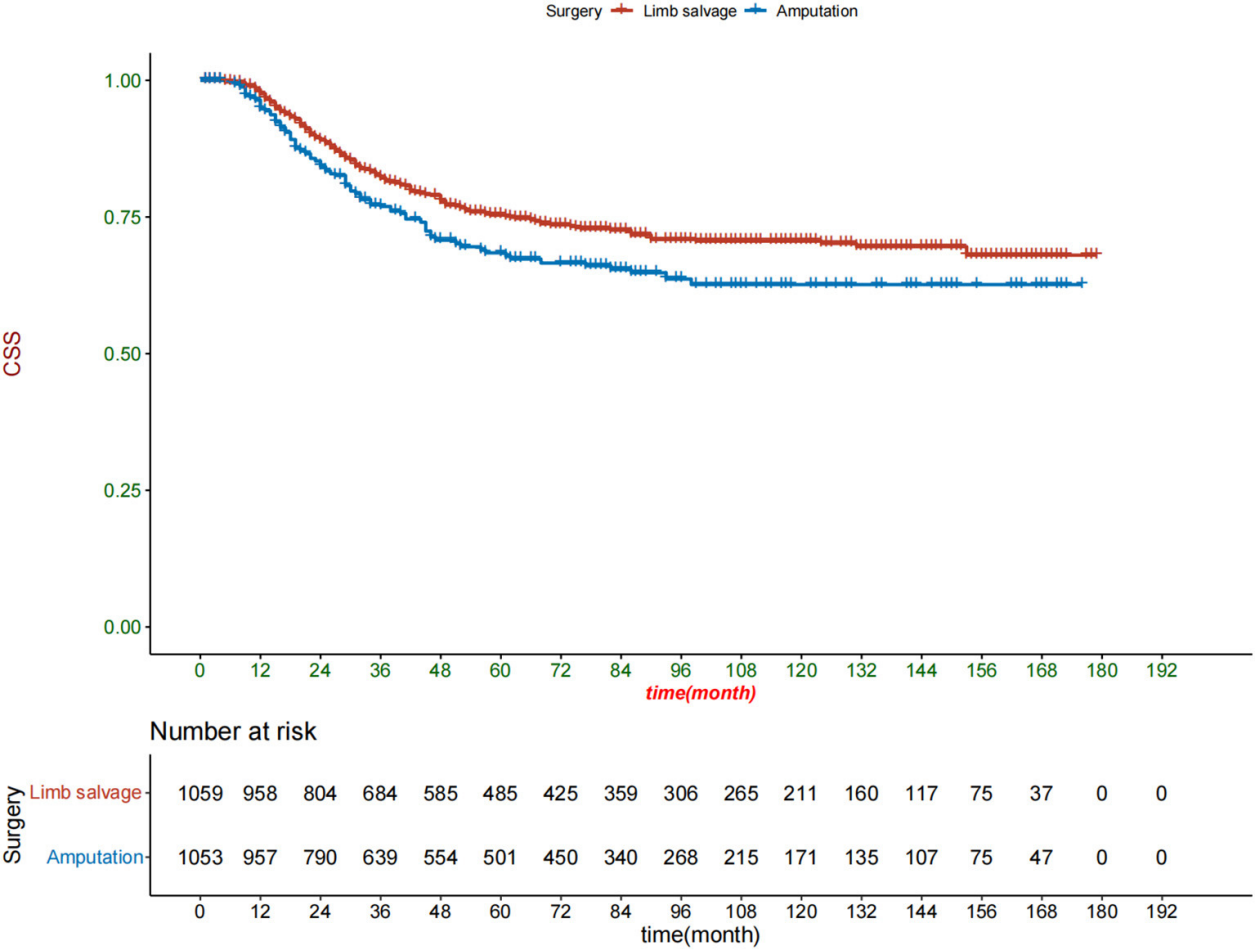
Kaplan-meier curve of patients receiving amputation and limb salvage after the inverse probability of treatment weighting matching.

After an optimized propensity matching analysis, a multivariable CRR model was used to compare CSM between limb salvage and amputation. (1) In the original cohort, amputation had a higher CSM than limb salvage (HR 1.4, *P* = 0.017); (2) There was no significant difference in CSM between amputation and limb salvage in the 1:2 cohort (HR 1.3, *P* = 0.065); but amputations still had higher CSM in the 1:4 cohort (HR 1.4, *P* = 0.017). (3) Patients who retained complete tumor grade information had higher CSM after amputation (HR1.4, *P* = 0.019). (4) We used inverse probability of treatment Weighting to match patients (Figure 5). After the inverse probability of treatment weighting, the Kaplan-Meier curve showed that patients who received amputation had a lower survival rate than those who had limb salvage (Figure 6).

## Discussion

In this study, amputation was found to have a higher CSM rate than limb salvage in children with limb osteosarcoma, similar to previous studies. However, compared with previous studies, this study included more patients and used PSM analysis to exclude confounding factors, resulting in more accurate results. The comparison between amputation and limb-salvage surgery was first studied by Simon et al. on 227 patients with Osteosarcoma and found that limb salvage surgery did not significantly improve the survival rate of patients (24). A subsequent analysis of 227 patients with Osteosarcoma at 26 institutions by Rougraff et al. (25) found the same results. The findings of Bacci et al. (26) also support the appellate results. However, with the development of multidisciplinary treatment and the maturity of prosthesis technology, the proportion of limb salvage surgery has been increasing (27). Recent studies have shown that limb salvage surgery significantly improves patient survival compared with amputation in patients with osteosarcoma (28–30). Although current osteosarcoma surgery tends to be limb salvage, previous studies have been small sample sizes and single-center studies, and few studies have specifically addressed pediatric Osteosarcoma. And the conclusions of previous studies may be influenced by confounding factors. Han et al. (20) conducted a meta-analysis showing that patients who underwent amputation had significantly lower survival rates than those who had limb preservation. But the heterogeneity between studies also leads to inevitable bias.

Moreover, there is another important outcome that we need to keep an eye on. Local recurrence of the tumor also puts the patient at risk for a second operation or amputation. Local recurrence is related to the tumor response to chemotherapy and the surgical margin, not the surgical method itself. Although limb salvage surgery has a higher survival rate, surgeons still prefer amputation for larger or more aggressive tumors. Still, some studies offer exciting news. Reddy et al. (15) analyzed 360 patients with Osteosarcoma and found that the survival rate of the second amputation after local tumor recurrence was similar to that of the first amputation. Grimer et al. (31) have also shown that patients with locally recurrent tumors can be cured by reoperation or amputation and radiotherapy. Therefore, limb salvage surgery remains the surgical option for pediatric osteosarcoma patients. However, amputation is still appropriate for stage IIB tumors that do not respond to chemotherapy, significant vascular and nerve tract involvement, and lack of bone or soft tissue reconstruction opportunities.

In this study, we focused on pediatric patients with Osteosarcoma, using the SEER database for a series of analyses. We used rigorous statistical methods, including propensity score matching and competitive risk models, to compare mortality differences between limb salvage surgery and amputation. Our study showed that girls are more likely to opt for limb salvage surgery, similar to a previous study (32). Previous studies have shown that tumor stage, tumor grade, and tumor size are all risk factors for the prognosis of Osteosarcoma and factors affecting surgical decision-making (30, 33). Limb salvage surgery has a higher survival rate than amputation, but this may be due to selection bias due to more aggressive tumors, such as neurovascular wrapping, poor response to chemotherapy, and tumor metastasis. In this study, the 5-year survival rate was 65.1% for patients with amputation and 76.5% for patients with limb salvage. However, these data have not been well recorded in the SEER database, and propensity matching cannot exclude confounding factors. Therefore, amputation is still recommended for highly invasive tumors.

In addition, the final endpoint of the patients was CSM. According to the SEER database, amputation has a higher CSM than limb salvage, and our results confirmed that patients with amputation have a 1.5-fold increase in CSM compared with patients with limb salvage. Further, we used various statistical methods to eliminate the differences caused by confounding factors. This included detailed adjustments for the patient's demographic information and underlying tumor characteristics using propensity matching. A competitive risk model was also used to adjust for the potential bias caused by OCM. We looked at the risk of death from amputation primarily in the 1:1 propensity matching cohort, and the same phenomenon was observed in the 1:4 matched cohort and the original cohort. In the end, the same results were found when unknown tumor grades were removed, confirming our concerns. Thus, amputation does indeed have a higher risk of death than limb salvage in pediatric osteosarcoma patients. Besides, we focused on OCM. Before matching, amputation had a higher OCM than limb salvage (HR3.4, *P* = 0.007), and there was no significant difference in survival between amputation and limb salvage after matching (HR3.2, *P* = 0.056). Thus, OCM was evenly distributed between amputees and patients with limb salvage, excluding differences in other causes of death. Since our study was a retrospective observational study, there were many data biases and confounding variables due to various reasons. We used PSM to reduce the impact of these biases and confounding variables.

Although our study used rigorous statistical methods, there are some limitations. First, this study was still retrospective, with no standardized specimen handling, lack of central pathological review, and no data on local recurrence and disease-free survival. Second, we also lack the patient's comorbidities information, cannot adjust the patient's other comorbidities. Although propensity score matching and the use of OCM to exclude comorbidities were performed to minimize bias, this is not equivalent to a prospective clinical randomized controlled trial. Finally, the SEER database does not contain information about the hospital, such as the level and capacity of the hospital. It does not include information about repeated treatments, such as surgery or radiotherapy after recurrence.

## Conclusions

Our results suggested that amputation is associated with a 1.5-fold increase in CSM in pediatric patients with limbs Osteosarcoma. Therefore, in the surgical selection of pediatric patients with Osteosarcoma, limb salvage surgery should be the first choice.

## Data Availability Statement

Publicly available datasets were analyzed in this study. This data can be found here: https://seer.Cancer.gov/.

## Ethics Statement

The data of this study is obtained from the SEER database. The patient's data is public and anonymous, so this study does not require ethical approval and informed consent.

## Author Contributions

JT, DH, and JW contributed to the conception, design, contributed to manuscript writing, and revision. CZ, JW, XT, TM, and JT collected and analyzed the data. ZZ, ML, XT, LJ, JW, and JT drew the figures and tables. JT and JW wrote the draft. All authors approved the final manuscript.

## Funding

Special Key Project of Chongqing Technology Innovation and Application Development (No. Cstc2019jscx-tjsbX0003).

## Conflict of Interest

The authors declare that the research was conducted in the absence of any commercial or financial relationships that could be construed as a potential conflict of interest.

## Publisher's Note

All claims expressed in this article are solely those of the authors and do not necessarily represent those of their affiliated organizations, or those of the publisher, the editors and the reviewers. Any product that may be evaluated in this article, or claim that may be made by its manufacturer, is not guaranteed or endorsed by the publisher.
